# Facing uncertainty in primary care: perceptions, strategies and educational needs – a qualitative explorative study with German general practice trainees

**DOI:** 10.1186/s12909-026-10550-x

**Published:** 2026-10-12

**Authors:** Andreas Christian Dreher, Pauline Scharli, Anika Mitzkat, Christine Becker, Attila Altiner, Simon Schwill

**Affiliations:** Department for Primary Care and Health Services Research Competence Centre for postgraduate medical education, University Hospital Heidelberg, Heidelberg, Im Neuenheimer Feld 130.3, 69120 Germany

**Keywords:** General practice, Primary care, Navigating uncertainty, Postgraduate medical education

## Abstract

**Background:**

Uncertainty is an inherent aspect of everyday practice in primary care with a context of low-prevalence conditions. Dealing with diagnostic ambiguity and therapeutic uncertainty constitutes a central challenge within postgraduate medical training in general practice (GP). This study aimed to explore how GP trainees perceive and manage uncertainty and their educational needs to tailor specific measures.

**Materials and methods:**

In 2025, GP trainees of the Competence Centre Baden-Württemberg (KWBW) were recruited by purposive sampling to participate in an interview-study. A semi-structured interview guide was specifically developed. All interviews were transcribed verbatim and subsequently analysed using a content-analytical approach by three researchers.

**Results:**

22 interviews with mean duration of 46 min (min. 36 min, max. 70 min) were performed. GP trainees experienced uncertainty regarding themselves (intrapersonal), their patients (interpersonal) and the primary care context (systemic). Common strategies to manage included watchful waiting, seeking collegial advice, consulting external sources and relying on experience and intuition. Uncertainty was more perceived in outpatient than in inpatient settings and was linked to various emotions. The interviewees recommend early and longitudinal integration of uncertainty management into training, as it represents a relevant competency in daily primary care.

**Conclusion:**

Navigating uncertainty appears a crucial skill, especially in primary care but current GP trainees have not experienced specific training. As a result, trainees often feel unprepared to manage it, which does not only affect outcomes of care such as quaternary prevention but also poses mental burden. Specific measures with longitudinal integration into postgraduate curricula may enhance trainees’ competency in navigating uncertainty and thereby could also reduce mental burden and support .

**Supplementary Information:**

The online version contains supplementary material available at https://doi.org/10.1186/s12909-026-10550-x.

## Introduction

Despite the desire for security and objectivity, medical practice is marked by uncertainty and intersubjectivity. Uncertainty is an inescapable part of everyday individualized patient-centred care [1]. Uncertainty is a complex and constant phenomenon of medicine and the focus of various research. Multiple valuable taxonomies exist, underlining the multidimensional nature of uncertainty in medicine: Beresford identified three different uncertainty sources: technical, conceptual and personal [2]. Hamui-Sutton distinguished technical, conceptual, communicational, ethical and systemic uncertainty [3]. Han classified uncertainty according to source, substantive issue and locus of uncertainty [4]. Purkl differs among biomedical, interpersonal and psychosocial uncertainty [5].

Uncertainty is common in all medical fields but with different extent: Specialist surgeons and internal medicine subspecialists reported less stress than generalists [6]. Primary care is characterized by its broad spectrum of unselected patients, its complexity and inherent uncertainty. General Practice (GP) is a clinical environment that accepts and works with uncertainty in difference from secondary or tertiary care [7]. The competence of dealing with uncertainty is essential for the quality of care in GP, as undifferentiated and unorganised problems are a daily challenge for GPs [8]. Affective intolerance of uncertainty is associated with increased diagnostic activity [9], which may harm patients through overdiagnosis or false-positive results in contrast to the purpose of quaternary prevention. Quaternary prevention aims to foster clinical decision-making that protects patients from medical interventions whose potential harms are likely to outweigh their expected benefits [10]. Overconfidence on the other side, is a proven cause of diagnostic error in medicine [11]. When starting to work in GP outpatient setting, the occurrence of uncertainty as a challenge is described [12, 13]. A low tolerance of uncertainty and ambiguity relates to reduced psychological well-being including higher burn-out rates [14]. In summary, uncertainty itself is not necessarily a problem in primary care. Rather the ability of GPs to tolerate and manage uncertainty, including recognising when it cannot or should not be eliminated, is an essential part of effective clinical practice [15]. Still, there is little evidence how uncertainty is managed in GP [16].

The imperative for teaching to navigate uncertainty effectively and purposefully is constituted by the certainty of uncertainty [17] and the acceptance that it can be reduced but never fully eliminated in medical practice [18]. This has impact on a full professional life as a physician and thereby longitudinally on undergraduate medical education, postgraduate medical training and continuous professional development: Lee et al. [19] developed a framework of uncertainty for medical education highlighting sources, subjective influencers, responses to uncertainty and the dynamic relationship between these elements. Han et al. [20] described that physicians manage clinical uncertainty through both strategies aimed at reducing uncertainty and strategies aimed at tolerating and mitigating its effects. They suggest that developing epistemic maturity, humility, flexibility and openness may enable physicians to accept uncertainty when it cannot or should not be eliminated. To improve uncertainty tolerance in medical students, problem-based learning, simulation and reflection have proven successful [21]. Ilgen et al. [22] presented educational strategies for managing clinical uncertainty including explicitly discussing uncertainty, distinguishing between uncertainty that can be reduced through further information and uncertainty that is inherent to clinical situations, and teaching trainees to use forward planning and monitoring rather than reflexively seeking to eliminate uncertainty through further interventions. GP trainees need to learn to manage the ambiguity inherent in the field as well as the uncertainty within postgraduate training. There is little empirical evidence about how GP trainees navigate the tension between reducing uncertainty through further investigation and accepting uncertainty as an inherent part of primary care. Exploring their experiences and perspectives can provide a more in-depth understanding of how uncertainty is managed in everyday practice and help identify educational needs for postgraduate training. One way could be education interventions addressing testing in primary care [23]. A review summarizes, that educators and instructors are pivotal to learn navigating uncertainty [7]. Still, specific measures for GP trainee’s as well as in continuous professional development appear scarce.

Therefore, this study aimed to explore how GP trainees perceive and manage uncertainty in primary care, including their strategies to reduce, tolerate and navigate uncertainty and how they communicate it with patients to assess educational needs for postgraduate training and further education.

## Materials and methods

### Type of study and setting

An explorative qualitative study by semi-structured online interviews with GP trainees was performed. Within a period of 14 weeks, subsequent recruitment, interviews and data-analysis had been performed from March to July 2025. Reporting is based on the consolidated criteria for reporting qualitative research (COREQ) (Supplement 1).

### Participants and recruitment

All interviewees were GP trainees and participants from the KWBW, the postgraduate medical training programme for GP trainees in Baden-Württemberg (federal state of Germany). In Germany GP training is five years full-time training structured by the medical association´s model speciality training regulations [24] with a 12-month in-patient rotation in internal medicine, a 24-month rotation in GP, a 6-month rotation in a third patient-centred medical discipline like pediatrics, psychiatry or dermatology as well as 18 months of rotation in further subdisciplines or internal medicine. The German postgraduate medical training could be described as an individual apprenticeship without compulsory institutional structured training. The KWBW Verbundweiterbildung PLUS© is the first German voluntary competency-based training program to offer a seminar curriculum including educational compact interventions as well as individual and group mentoring, train-the-trainer courses and the possibility of regional clinical rotations across Baden-Württemberg for GP trainees [25, 26]. Most participants attend approximately 24 teaching units per year within comprehensive seminars on primary care topics and some participate in the group mentoring and the local rotation planning. While some GP trainees participate throughout their entire postgraduate training, others join the programme only during the two-year general practice rotation at the end of their training. Out of all trainees (*N* = 651), we recruited participants using purposive sampling: Firstly, trainees’ current rotation needed to be in GP clinics. Furthermore, trainees were differentiated into those, who planned to become a GP right after graduation from medical school (direct entry), those who switched their training after some years of postgraduate training elsewhere (advanced entry) and those, who already had finished another specialisation before (lateral entry) [27, 28]. Trainees were contacted via mail. No approached trainee declined to participate and no participants withdrew from the study.

### Development of instrument, interview guide

After literature-research (NIH/PubMed^®^: „uncertainty“, „general practice/family medicine“, „residency“, „education“) an interview guide was developed by an interprofessional group of four researchers (ACD, PS, AM, SS) experienced in qualitative research and primary care. It was first validated using think-aloud technique [29]. In this method, participants verbalize their thought process whilst completing the interview, providing insights into their cognitive strategies and decision making. Secondly, the interview guideline was pre-tested with two interviewees leading to minor changes before it was finalised (Supplement 2). Questions included experience of uncertainty, dealing with uncertainty, communicating uncertainty, previous education in uncertainty and educational needs.

### Data collection

Interviews were conducted via Zoom^®^ (San Jose, California USA, Version 6.4.6) by either ACD (board certified specialist in Psychosomatic Medicine and actual GP trainee in the final year (part-time) and researcher (part-time), experienced in qualitative research) or PS (GP trainee in the second year (part-time) and researcher (part-time), beginner in qualitative research). The interviewees were not known to the interviewers. The researchers introduced themselves, explained their role and presented the research questions. Interviews were conducted at the workplace or in the researcher’s home. No other persons (non-participants) were present during the interviews. Field notes were taken throughout the data collection process to document relevant observations. The transcripts were not provided to the participants for correction.

### Qualitative data analysis

Audio recordings of the interviews were transcribed verbatim and analysed paper based. Qualitative data was analysed using qualitative content analysis following Kuckartz [30] by two independent researchers (ACD, PS) independently. Main categories were derived deductively from the research questions and the interview guide. During the analysis, additional categories and subcategories were added inductively based on the interview material. Categories and quotes were discussed and adapted if needed until consent concerning categorisation was reached. In case of missing consent, a third scientist (SS, board-certified GP (part-time), Master of Medical Education, leading researcher (part-time), very experienced in qualitative research) was consulted. The coded material was then compared within and across categories to identify commonalities and differences relevant to the research questions. Throughout the analytical process, continuous reflexivity was maintained regarding personal, interpersonal, methodological and contextual factors that might have influenced data protection [31].

## Results

Among 22 interviewed GP trainees, uncertainty emerged as a multidimensional experience encompassing intrapersonal, interpersonal and systemic domains. Trainees described uncertainty as an inherent aspect of general practice, with experience, supportive learning environments and reflective approaches facilitating its management and contributing to professional identity formation in general practice.

### Interview process

A total of *n* = 22 interviews were conducted between March 2025 and July 2025 with an average duration of 46 min (ranging from a minimum of 36 min to a maximum of 70 min). The interviews were conducted without any technical issues. No interview had to be repeated. No new themes or codes emerged in the final interviews, indicating that meaning saturation had been achieved and no additional participants were recruited.

### Sociodemographic data

Sociodemographic data is shown in (Table 1). All trainees worked in the GP´s practice (outpatient care). Seven trainees were in direct entry, 8 trainees in advanced entry and 7 in lateral entry (two specialists in anaesthesiology, two specialists in internal medicine, one specialist in gynaecology, one specialist in radiology and one specialist in orthopaedics). The mean duration of training in GP participants already underwent by the time of the interview was 1.2 years.

**Table 1:** Sociodemographic data of General Practice trainees participating in the interview study (*N* = 22)

*n* = 22		total	Direct entry	Advanced entry	Lateral entry
Sex	Female	17 (77.3%)	4 (57.1%)	8 (100.0%)	5 (71.4%)
	Male	5 (22.7%)	3 (42.9%)	0 (0.0%)	2 (28.6%)
Age (years)	Median (IQ)	36 (32;40)	33 (31;36)	36 (32;40)	42 (39;45)
Year of training	average	4.0 (SD = 1.31)	3.0 (SD = 1.85)	4.5 (SD = 0.5)	4.4 (SD = 0.49)
Duration of GP rotation	years (SD)	1.2 (SD = 0.53)	1.1 (SD = 0.46)	1.5 (SD = 0.49)	1.1 (SD = 0.56)
Location of GP rotation	urban	12 (54.6%)	6 (85.7%)	2 (25%)	4 (57.1%)
	rural	10 (45.4%)	1 (14.3%)	6 (75%)	3 (42.9%)

### Perception of uncertainty

Trainees described experiences of uncertainty which were categorised in three domains: Intrapersonal, interpersonal, and systemic uncertainty (Table 2). Intrapersonal uncertainty is the uncertainty arising within the GP trainee. Interpersonal uncertainty is the uncertainty arising in interactions between the GP trainee and other people. Systemic uncertainty is arising from the healthcare system and the context in which the GP trainee works. Intrapersonal uncertainty was reported at multiple levels, including cognitive (uncertainty as a lack of competence), affective (fear of mistakes, insecurity) and ethical level (obligation to disclose uncertainty). Trainees highlighted their own tolerance of uncertainty varying for example with diagnostic ambiguity, prognostic unawareness and decision without full information. They also described a personal learning process in differentiating between professional and personal uncertainty

**Table 2:** Categories of uncertainty mentioned by General Practice (GP) trainees categorized in intrapersonal, interpersonal and systemic uncertainty (*N* = 22)

Intrapersonal uncertainty	Cognitive aspects	*„For me*,* uncertainty is an actual or perceived lack of competence.“ (#17)* *“For me*,* uncertainty is not a bad thing in this sense*,* but rather something that spurs me on to discuss something with other disciplines or in my private life*,* or to read up on it again.” (#19)*
	Affective aspects	*“I’m also a little afraid of overlooking something or not doing justice to it*,* or yes*,* of doing something wrong*,* making mistakes. Insecurity is also a feeling. (#13)*
	Tolerance of uncertainty	*„that I feel so tense (when I have to tolerate uncertainty) that I know*,* or tell myself afterwards*,* that it’s not uncertainty arising from a lack of competence*,* but from the situation itself.“ (#20)* *„Well*,* I’m more of a double-checker.“ (#17)*
	Ethical concept	*“I could imagine that [the challenge] for some people is communicating their own insecurity*,* but for me personally*,* I find it a relief and just ethical.“ (#2)*
	Learning progress	*“Then there is professional uncertainty and interpersonal or personal uncertainty. Professional uncertainty arises when I am not in my comfort zone professionally*, i.e.,* when I am not entirely sure whether I can offer the right solution or interim solution. Or whether I am overlooking something relevant. (#3)*
Interpersonal uncertainty	Patient - doctor	*“When it’s simply not clear where the journey is headed*,* when patients report a lot of things*,* sometimes there’s no common thread*,* at least from the patient’s perspective*,* which means you don’t really know exactly what the requirement is.” (#1)* *„Endure uncertainty*,* depending on whether it belongs to your perception above or to your interaction here: I was nervous*,* I had the feeling that certain emotions might have been transferred to me by each patient.”(#6)*
	Resident - trainer	*“She (my trainer) then kind of backtracked a little with him*,* with the therapy I had just started with him. And that didn’t really unsettle me professionally*,* but rather personally.” (#16)*
	Doctor – other specialities	*„that you have a little bit of self-doubt as to whether the patient is in good hands with me as their family doctor or whether they would receive better treatment from a specialist.“ (#22)*
	Doctor - other	*„Interpersonal uncertainty arises when I feel provoked or threatened or outside my comfort zone.”(#3)*
Systemic uncertainty	Biomedical	*„Patients often do not present with classic medical symptoms and diseases. So I find it almost easier to make a pathological diagnosis and draw conclusions from it than to have a vague feeling that something is wrong but not being able to pinpoint it.“ (#17)*
	psychosocial	*„No consultation without a question mark at the end: what else could be medical*,* what psychosocial factors or unknown factors are at play here?“ (#10)*
	Evidence based medicine	*“It’s a mixture of intuition*,* but sometimes also based on collected examination values*,* where I then say*,* okay*,* now I’m going to do something different than what is currently stated in the guideline*,* which does not accurately reflect my patient’s situation.”(#2)*
	Primary care setting	*“That I currently have no indication of any acute danger*,* for example*,* and that we will leave it open for now and wait to see how things develop. But I will try to reflect this or be open about my uncertainty […]*,* which does not mean that I am leaving you alone with this*,* but rather that we simply need to observe the situation together for a while and then meet again.” (#5)* *As a general practitioner*,* I can’t know everything. (#19)*
	Error unfriendly matrix	*„Medical errors are sensationalized in the media*,* prosecuted*,* and there is no space where they can be discussed.“ (#6)*
	Other / further	*“Maybe you’re having a bad day too*,* then [the uncertainty] is even worse. Or you had a difficult patient before […]. It all plays a part. Sleep deprivation with small children.” (#4)*

Interpersonal uncertainty arose in interactions with patients, trainers and colleagues from other specialties. Trainees described difficulties in situations where patient narratives lacked clarity, where trainer action or feedback conflicted with their decisions, or when they questioned whether patients might be better cared for by specialists due to competence limits. Interpersonal challenges also extended to feelings of being provoked or unsettled in professional encounters

Systemic uncertainty referred to a wider medical and social context. Trainees emphasised the complexity of biomedical and psychosocial presentations, limitations of evidence-based guidelines and the realities of primary care where not every problem could be solved immediately. They also criticized an error-unfriendly culture within the healthcare system, leading to forensic actions.

Trainees stated a higher perceived level of stress due to uncertainty in outpatient care than in inpatient care due to patients with unspecific symptoms and multimorbidity whilst limited access to fast special diagnostic tests and exchange with other disciplines. They stated that uncertainty is also inherent in the clinical setting but other strategies of dealing with it are likely to be applied.

*“[In the hospital]*,* the goal was to minimize uncertainty as much as possiblewith relatively extensive diagnostic efforts […] Of course*,* this is not practical*. *for patients or the general healthcare system in an outpatient setting.” (#22)*.

Participants described dealing with uncertainty as an important part of developing their professional identity as GPs and getting to know their professional role in the healthcare system. They emphasised that acknowledging uncertainty and accepting the limits of their own knowledge were associated with humility and could strengthen their confidence as GPs. At the same time, uncertainty was perceived not only as a challenge but also as an inherent and intellectually stimulating part of general practice that requires developing individual strategies and trusting one’s clinical judgement.

*“[It is] also somewhat about your self-confidence as a GP*,* [in that] the way you deal with uncertainty can strengthen your self-confidence.”* (#10).

### Handling of uncertainty

Trainees employed a broad range of strategies to navigate uncertainty (Table 3), including collegial exchange and referrals, the use of validated external resources, watchful waiting, sharing uncertainty with patients or interprofessional networking. Participants perceived that increasing experience in GP helped them to use intuition more in dealing with uncertainty, whilst experience and intuition seem to control each other. Trainees reported that a constructive error culture enables a safe exploratory learning space for evidence-informed handling of the inherent uncertainties of primary care.

**Table 3:** Strategies for navigating uncertainty by General Practice (GP) trainees (*N* = 22)

Collegial exchange	supervision (trainer) intervision with other GPs intervision across specialities reflection in Balint groups
Individual reflexion	taking time-out for reflection drawing on professional experience use of intuition
Knowledge-based strategies	looking up guideline recommendations looking up information in validated sources conducting further diagnostics referral to specialists
Patient-centred strategies	patient as partner („shared uncertainty“) watchful waiting (incl. yellow/red flags) scheduling fixed follow-up appointment proactive monitoring of clinical course

### Communicating uncertainty

Trainees have a high interest in communicating uncertainty taking the capability of patient´s health literacy into account but feared it because it may also be perceived as incompetence.

*“For example [I can communicate with a patient: ]*,*” I currently have no indication of any acute danger*,* so we’ll leave it open for now and wait to see how things develop”. But I’ll try to reflect that or be open about my uncertainty […]*,* which doesn’t mean leaving [the patient]alone with it*,* but rather that [me and the patient] just have to observe it together for a while and see how things develop.” (#*5).

### Training of handling uncertainty

Representative quotes about previous educational experiences, educational needs for medical school and postgraduate medical training in GP can be found in (Table 4).

**Table 4:** Representative quotes about previous educational experience with learning to navigate uncertainty and educational needs for undergraduate and postgraduate medical education (*N* = 22)

Previous Educational Experience	“At the university hospital, however, it is sometimes suggested that there is no [uncertainty]; diagnostics are performed until there is no longer any uncertainty.” (#10) “This subject-specific learning, as well as university learning and teaching, has left little room for uncertainty to be part of the process.” (#18) „Well, I think that at least in my days, (uncertainty) was a no-go, and some students can’t even imagine that after taking a medical history, performing an examination, and taking lab samples, you still don’t know what’s wrong with the patient.“ (#19) “On a purely statistical level or in terms of prevalence, [but] it remained somewhat nebulous as to how this intuition should arise. […] I do think that the code has always been that one should at least project confidence to the outside world.” (#20)
Educational Needs for undergraduate medical education (medical school)	*“Admit that we are human beings and that in medicine there really aren’t many things that are always 100% the same.” (#8)* *„From the outset and in all subjects and courses as a “school of thought”: bias (e.g.*,* availability bias)*,* error culture*,* uncertainty*,* etc.“ (#11)* *“That you do these [role-playing games] yourself in groups*,* where you specifically address this issue. There are techniques and phrasing aids that don’t necessarily hide your uncertainty but make you appear a little more confident.” (#16)* *“I think a certain amount of training can be incorporated. It has to do with strengthening personality and personal development. To say that there are uncertainties*,* that this is a normal mistake*,* that a culture of mistakes is part of life*,* that mistakes are inevitable*,* that mistakes will happen.” (#17)* *„So there are already techniques and phrasing aids that don’t hide your uncertainty but make you appear a little more confident.“ (#18)*
Educational Needs for postgraduate medical training (specialisation)	*“An open ear from the trainers*,* perhaps even a training discussion on the topic of dealing with uncertainties.” (#4)* *“Perhaps a bit like a Balint group*,* where you simply have a setting where you can present cases again and then discuss not only what would have been medically right or wrong*,* but also a little bit about why I’m so unsure about it.” (#12)* *“Uncertainties about trivial matters also exist*,* and it is important to discuss these too with the trainers.”(#19)* *“I think it’s important to see that other people also have insecurities*,* and I think it’s good to learn (in seminars) how to deal with them and also to receive communication training on this topic.”(#20)* *“I find it most interesting and helpful when people share their experiences and even someone with 20 years of professional experience admits that they still encounter situations where they feel uncertain. And this applies to family doctors*,* professors and specialists alike.” (#21)* *“And the challenge is then also that when a mistake happens*,* of course*,* you have to deal with the mistake sensibly (…) to have a good error culture.” (#22)*

Trainees reported little teaching and highlighted a gap between training and the realities of medical care. In medical education, uncertainty appeared rather discrete than addressed explicitly. GP trainees regret this, because of its centrality in daily GP. Several participants reported a tendency to deny uncertainty within medical education. While simplification was considered useful for disease-specific learning in medical education, it was perceived as insufficient preparation for the complexity and uncertainty of clinical practice. Most trainees described a personal process of learning to accept uncertainty as an inherent part of medicine, which led them to seek strategies for managing and communicating it. They emphasized the need for uncertainty to be addressed earlier, more openly and continuously throughout medical training. For developing competence in handling uncertainty during residency, they highlighted the importance of supportive supervision (also regarding seemingly trivial uncertainties), a constructive error culture, opportunities to share experiences with peers and senior colleagues and structured formats such as communication training and reflective seminars. With increasing experience, trainees more strongly integrated the competence of dealing with uncertainty into their professional identity.

“Dealing with uncertainty shapes me not only as a general practitioner, but also, as a person. Despite all the evidence-based approaches, which I find important and wonderful, one must always consider that these are only probabilities that may not apply to individuals.” (#6)

## Discussion

This qualitative study highlights the pervasive character of uncertainty in daily medical care, its practical significance and the educational challenges it poses for GP trainees and trainers. The study found a clear gap between the central role of uncertainty in everyday GP and its limited role in formal medical education. GP trainees described uncertainty as intrapersonal, interpersonal and systemic and reported using different strategies to manage it, including seeking advice, referring patients, watchful waiting and communicating uncertainty with patients. They described a clear need to address uncertainty more explicitly and continuously during postgraduate medical training, with supportive supervision, opportunities for reflection and peer exchange and a constructive error culture.

Furthermore it showed that education in navigating uncertainty is complex and does not evolve spontaneously but should be embedded into training. The contribution of this study lies in integrating trainees’ perceptions, management and communication of uncertainty with their prior educational experiences and expressed educational needs. All the objectives of this study were achieved.

### Navigating uncertainty

There was a strong agreement, that uncertainty plays a highly relevant role in GP postgraduate training. The high prevalence and relevance of uncertainty in GP is also described in earlier studies [8, 9].

Trainees at the beginning of their GP rotation reported higher levels of stress related to uncertainty and more likely associated it with perceived personal incompetence and tended to higher diagnostic and referral activity. With increasing experience, trainees described less stress and demonstrated a more differentiated reflexion, recognising a broader range of sources of uncertainty and taking further intrapersonal factors into account. The relevance of uncertainty when starting in GP was also described earlier [12, 13]. Peek et al. [32] revealed a positive association between tolerance of uncertainty and thriving and a negative association between tolerance of uncertainty and perceived stress. This aligns with results from controlled laboratory settings revealing a positive association between tolerance of uncertainty and performance in decision-making in conditions of uncertainty [33, 34]. Moreover, a relationship between low tolerance of uncertainty and ambiguity and mental disorder is known [35], which is also shown in medical trainees [14]. Previous studies have shown that GP trainees considerably suffer from stress with higher prevalence of depressive mood [36]. The competency of dealing with uncertainty does not only reduce mental burden, it also fosters and enables efficient learning and high-quality care. For instance, Schneider et al. [9] described a connection of higher diagnostic activity in case of low tolerance of uncertainty.

We could also see a tendency, that with more experience in GP they got more used to managing uncertainty and less aversive affects were connected with it. Uncertainty was more accepted and integrated into critical thinking and communication whereas with less experience the wish to reduce uncertainty seemed higher. It was not automatically understood as something adverse [5], but it was connected with aversive feelings that did not change with more experience but were less intense and better to tolerate. With increasing experience uncertainty was seen as a diagnostic resource and challenge to research for new information and to reflect on own decisions and behaviours. Ilgen et al. [37] suggest that clinicians can use discomfort as a signal of uncertainty, prompting reflection and deliberate adjustment of their clinical reasoning rather than simply trying to eliminate uncertainty. GP-specific interventions like watchful waiting and safety-netting with follow-up appointments were seen within all trainees, whilst trainees with more experience were more likely to trust this procedure as professional, correct and enough. All trainees referred to shared decision [38] making as the overarching concept for communicating uncertainty, which can be understood as “shared uncertainty”. Patients unused to „shared uncertainty“ may react with irritation. The professional challenge lies in conveying uncertainty while maintaining credibility. Therefore, change of culture also requires patients accept reflecting uncertainty as a professional and transparent approach.

Our findings support that learning to live with uncertainty is part of developing a professional identity in GP. Participants described acknowledging the limits of their knowledge, accepting uncertainty and developing individual ways of dealing with it as aspects of becoming a confident and professional GP. Most likely, learning to deal with uncertainty is an integral part of professional development and identity formation in GP trainees. PIF in medical education is getting more attentions within the last 15 years [39]. Professional identity formation is a dynamic process influenced by a broad range of interacting individual, social, educational and clinical factors, extending from personal experiences and values to socialization within the learning environment [40, 41]. In a recent study, Schwill et al. published a tool to assess socialisation of GP trainees [42].

The professional identity of general practitioners is characterized by the provision of personal, primary, preventive, comprehensive, continuous and coordinated care, addressing individuals within the broader contexts of their families, communities and environment. In a current review, Tsang et al. [43] showed the importance of specialty-specific characteristics in shaping professional identity formation and criticised a lack of focus on PIF in GP-training and suggested explicit attention to PIF in three key areas: curriculum, faculty development and teaching and learning. Taking uncertainty as a part of PIF in GP a similar didactic integration into GP training seems a logic consequence. Regarding teaching and learning, mentoring and role modelling as well as reflective teaching approaches [39, 44, 45] may play a key role in integrating managing uncertainty into professional identity formation, particularly by enabling learners to reflect constructively on uncertainty.

### Train to navigate uncertainty

In line with Kern´s six -step approach [46] we explored prior learning experiences, current educational needs and preferences for didactic methods. This framework supports the alignment of our findings with our own curriculum improvement. Recommendations for training to manage uncertainty are described for a new understanding of uncertainty in medical education [47]. To improve uncertainty, tolerance in medical students, problem-based learning, simulation and reflection have proven successful [21], which correlates with the main educational needs of the interviewees. The transition from medical student to GP trainee itself relates to uncertainty and achieving postgraduate work readiness is a goal of undergraduate education [48, 49]. The competence of managing uncertainty should be one for work readiness and was missing according to our interviewees.

Since uncertainty encompasses intrapersonal, interpersonal and systemic dimensions, it constitutes a complex phenomenon and specific measures to train navigating uncertainty should focus all three dimensions. At KWBW, educational compact interventions have proven successful [26]. One such intervention, carried out at the start of the training programme, addresses the core values and principles of GP [50], including reflection on uncertainty and GP-specific work principles as practical strategies for navigating uncertainty. In future, the intrapersonal dimensions of doctors can be reflected in one-to-one mentoring or mentoring groups, Balint groups [51] and supervision. Interpersonal dimensions can be reflected upon and developed through communication training seminars involving simulation-based training, role-play and innovative techniques such as virtual reality, which incorporates feedback loops. There are already successful research works and communication trainings for uncertainty increasing diagnostic and clinical excellence [52]. Interpersonal dimensions can also be explored within Balint groups, intervision with peers and supervision. Systemic dimensions should form part of seminars, self-study with guidelines and training under supervision in an error-friendly training environment and could be discussed during intervision or mentoring.

We think that longitudinal curricula focussing professional development including self-reflexion and meta-reflexion of professional actions, with educational compact interventions at the beginning and corresponding further learning objectives, can also be effective in other countries and disciplines of medicine and non-medical fields where uncertainty is commonplace. We suggest integrating such longitudinal curricula into existing curricula at both pre- and post-graduate levels. Further educational implementation may involve integrating a spiral curriculum, practice- and case-based learning, and encouraging longitudinal reflection on intra-, interpersonal, and systemic dimensions [53, 54].

### Strengths and limitations

This qualitative study provides insight into subjective experiences and coping strategies with a good number of participants. The interviewees were trainees in Baden-Württemberg, a federal state of Germany with approximately 11.2 million inhabitants. Their views may be representative of the whole of Germany but not fully representative for GP trainees in general due to potential health system-specific, cultural and linguistic limitations. As most aspects about dealing with uncertainty are also described in international literature, we believe that educational implications are applicable to education in other countries. The exploratory study design is not intended to be exhaustive. The recruitment process was designed to capture the diversity of GP trainees as fully as possible. The participants in this study may have been more open-minded and empathetic than the average GP trainee. Navigating uncertainty may become another dimension which could be assessed to explore PIF in GP and visualise longitudinal progress Future research should focus on mixed-methods designs, integrating scales regarding uncertainty tolerance and professional identity formation with longitudinal date, which could explore developmental process and intervention effects more systematically.

## Conclusion

Navigating uncertainty appears a crucial skill, especially in primary care but current GP trainees have not experienced specific training. As a result, trainees often feel unprepared to manage it, especially at the beginning of specialisation and when starting rotation in primary care. While uncertainty can aid clinical reasoning, it also poses a mental burden. Measures should focus on intrapersonal, interpersonal and systemic dimensions of uncertainty and require an error-friendly environment at the training post. Specific measures with longitudinal integration into postgraduate curricula may enhance trainees’ competency in navigating uncertainty and thereby could also reduce mental burden and support professional identity formation.

## Supplementary Information

Supplementary Material 1.

Supplementary Material 2.

## Data Availability

The datasets presented in this article are not readily available because as required by the European Data Protection law the participants were informed of the privacy policy and agreed that the pseudonymized data would be available to the project team and would be stored at the study center. Request to access the datasets should be directed to Simon Schwill (simon.schwill@med.uni-heidelberg.de).

## Abbrevations

GP: General Practice.
