# Supplementary material for: Facing uncertainty in primary care: perceptions, strategies and educational needs – a qualitative explorative study with German general practice trainees
Source: BMC Med Educ. 2026 Oct 12;26:1627. doi: 10.1186/s12909-026-10550-x (PMC13659999; doi:10.1186/s12909-026-10550-x)
Supplement: Supplementary file 1 [file 12909_2026_10550_MOESM1_ESM.docx]

**navigating uncertainty**

**date/#:**

**Interview guideline**

Research questions:

**Navigating uncertainty:**

How do GP trainees perceive uncertainty and how do they deal with it?

*Can epistemic constructs be explored?*

How do GP trainees communicate uncertainty with patients?

Which implications for medical education and postgraduate training can be explored?

1) **Has the interviewee been informed about the study and has the consent form been signed?**
2) **Before starting the interview, we would kindly ask for the following sociodemographic information**

| *Age, gender* |  |
| --- | --- |
| *Professional career history* | How did you get into General Practice (direct entry / advanced entry / lateral entry)  Year of training  How long do you already work in General Practice?  Where is your training placement located? (urban/rural) |

3) **Then we would begin recording the interview:**

| **Topic** | **question** | **time** |
| --- | --- | --- |
| Consent  Thank you for agreeing to participating in this interview / study. Before starting recording, I would like you to confirm, that you have received the information sheet, that all your questions about the study have been answered and that you consent to the interview being recorded on audio. Please answer yes or no. | | 1 min  **START** |
| **Ice breaker** | How did you come to the Competence Center? | 4 Min |
|  | In our daily work as GPs, we face often situations, where we are confronted with uncertainty. |  |
| **Perception of uncertainty** | How do you experience uncertainty in General Practice? (open question!)  Can you think of examples, where you experienced uncertainty?  What is uncertainty from your point of view?  Which feelings do you have about uncertainty? | 5 min |
| **Coping and dealing with uncertainty** | Which strategies do you use to deal with uncertainty?  Which challenges come to your mind regarding dealing with uncertainty?  How do you decide under the circumstance of uncertainty?  (if now answer: for example further diagnostics or watchful waiting)  Have you ever found yourself in a situation where you had to tolerate / endure uncertainty? Tell me more…  How did you cope with it?  How did this feel for you?  To what extent does your professional experience influence how you deal with uncertainty?  Which role plays intuition how you deal with uncertainty? | 5 min  5 min |
| **Differences inpatient / outpatient care** | Have you noticed difference in dealing with uncertainty between inpatient and outpatient care? | 2 min |
| **Communicating uncertainty** | Do you communicate uncertainty with patients and if yes, how?  What is your impression, how patients feel and think about this?  For which patients do you consider communicating uncertainty as particularly helpful?  (if not already mentioned: Which role plays shared decision making in consultations with the circumstance of uncertainty?) | 5 min |
| **Best Practices** | Which Best Practices for dealing with uncertainty do you know and did you experience helpful? | 2 min |
| **Need in General Practice** | What would you need specifically for dealing with uncertainty in General Practice? | 2 min |
| **Education** | Was uncertainty an issue / topic during medical school?  If yes, where and how?  **Assuming you were to plan and conceptualize a course on this topic: How do you think students can be taught to deal with uncertainty (whilst still at university)**  What would you like for GP training? | 5 min |
| **Professional Identity** | How does uncertainty affect your professional identity and your view of yourself as a GP? | 2 min |
| **Coming to the end**  **(preparation)** | We are soon finished. Would you say that everything relevant to this topic for you was said during the interview? Do you want to add something to this topic? | 2 min |
| **END** | **We would stop recording now and thank you very much for your participation.** | 1 min |
