# Supplementary material for: Facing uncertainty in primary care: perceptions, strategies and educational needs – a qualitative explorative study with German general practice trainees
Source: BMC Med Educ. 2026 Oct 12;26:1627. doi: 10.1186/s12909-026-10550-x (PMC13659999; doi:10.1186/s12909-026-10550-x)
Supplement: Supplementary file 2 [file 12909_2026_10550_MOESM2_ESM.docx]

**Facing uncertainty in Primary Care: perceptions, strategies and educational needs – a qualitative explorative study with German General Practice trainees**

**COREQ**
